# A Selection from the Most Esteemed Treatises, Monographs, Prize Essays, Dissertations, and Cases Illustrating the Diseases of Women, and the Peculiarities of Pregnancy and the Puerperal State

**Published:** 1842-10

**Authors:** 


Art. VI.—*Analekten für Frauenkrankheiten, etc.—Leipsig,* 1837-41. 8vo, 10 Hefte.

*A Selection from the most esteemed Treatises, Monographs, Prize Essays, Dissertations, and Cases illustrating the Diseases of Women, and the Peculiarities of Pregnancy and the Puerperal State.—Leipsic,* 1837-41. 10 Numbers.

The title of the work explains its nature, which is analogous to that of the Selections on Children's Diseases, of which a notice recently appeared in this Journal. The papers it contains are generally well chosen, and among them are several very valuable essays on puerperal fever by continental and British writers. After our recent review of Heim and Kiwisch, however, we will not again enter on the subject of puerperal fever, and a great number of the other papers being translated from the English would present little that is novel to our readers. The work, however, which is not yet completed, will be found a valuable addition to an obstetric library.

